# Steep topography buffers threatened gymnosperm species against anthropogenic pressures in China

**DOI:** 10.1002/ece3.5983

**Published:** 2020-02-05

**Authors:** Ditte Arp Jensen, Keping Ma, Jens‐Christian Svenning

**Affiliations:** ^1^ Center for Biodiversity Dynamics in a Changing World (BIOCHANGE) Department of Bioscience Aarhus University Aarhus C Denmark; ^2^ Section for Ecoinformatics and Biodiversity Department of Bioscience Aarhus University Aarhus C Denmark; ^3^ Sino‐Danish Center for Education and Research (SDC) Water and Environment Programme, Eastern Yanqihu Campus University of Chinese Academy of Sciences Beijing China; ^4^ State Key Laboratory of Vegetation and Environmental Change Institute of Botany Chinese Academy of Sciences Beijing China

**Keywords:** anthropocene, China, gymnosperms, human population, species distributions, topography

## Abstract

China is one of the most species‐rich countries in the world, harboring many rare gymnosperms. Following recent human‐led loss of forests, China is now experiencing increases in forest cover resulting from efforts of reforestation schemes. As anthropogenic activities have previously been found to interact with topography in shaping forest cover in China and considering the large human population and the ongoing population increase of the country, it is important to understand the role of anthropogenic pressures relative to environmental drivers for shaping species distributions here. Based on the well‐established relationship between human population density and topography, we propose a hypothesis for explaining species distributions in a country dominated by human activities, predicting that species are more likely to occur in areas of steep topography under medium human population densities compared to low and high human population densities. Using species occurrence data from the Chinese Vascular Plant Distribution Database along with a common SDM method (maximum entropy modeling), we tested this hypothesis. Our results show that steep topography has the highest importance for predicting Chinese gymnosperm species occurrences in general, and threatened species specifically, in areas of medium human population densities. Consequently, these species are more often found in areas of steep terrain, supporting the proposed hypothesis. Results from this study highlight the need to include topographically heterogeneous habitats when planning new protected areas for species conservation.

## Graphical abstract

Topography and human activity have previously been found to affect forest cover and mammal distributions in China. Here, we test the hypothesis that gymnosperm species are more associated with steep topography in regions of medium human activity. Our results show a significantly higher importance of topography for predicting species occurrence at medium human population densities, supporting this hypothesis and highlighting the need to include topographically heterogeneous habitats when planning new protected areas for species conservation.

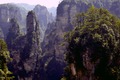

## 1 INTRODUCTION

The global human population strongly increased across the 20th century and is projected to further increase through the 21st century (Crutzen, 2002; UN/DESA, 2017). China has a long and well‐known history of dense human settlement dating back around 4,000 years. In recent times, however, China has experienced a rapid population increase unparalleled anywhere else in the world. From 1950 to 2018 alone, the Chinese population increased from 550 million to 1.4 billion (worldometers.info), making it the world's most populous country.

China's large population increase and the associated development in agriculture and infrastructure have led to ecosystem degradation and deforestation, putting many species under severe pressure and causing a strong need for conservation and restoration efforts (Isbell et al., 2017; Li, 2004). One of the main drivers of deforestation in China has been the demand for wood products, both for national use and for export (Wang, Innes, Lei, Dai, & Wu, 2007). The decline of forests in China has been recognized since the 1950s, and policies have been implemented to counteract this development, albeit their effectiveness has been debated (Robbins & Harrell, 2014). In 1998, following a series of destructive floods in southwestern China, resulting from logging operations beginning in the 1950s, new national‐scale reforestation initiatives were introduced. The Natural Forest Protection Program (NFPP) and the Returning Farmland to Forest Program have since cooperated in protecting and restoring forests, through logging bans and plantations in many parts of China (Robbins & Harrell, 2014; Wang et al., 2007). Due to these policies—as well as socioeconomically driven land abandonment—China's forest cover increased from 8.6% in 1949 to 20% in 2003 (Liu, Liu, Chen, & Long, 2010; Robbins & Harrell, 2014; Zhang & Song, 2006), although mostly by species‐poor plantations that do not represent the same level of biodiversity as natural forests (Hua et al., 2016). Despite the increasing focus on environmental protection, many plant species in China are continually threatened by human activities such as logging, harvesting of wild plants, and agricultural development (Volis, 2016).

Besides being the world's most populous country, China is also one of the most species‐rich countries in the world, containing around 33,000 species of vascular plants whereof almost half are endemics (Huang, Chen, Ying, & Ma, 2011). Many of these endemics are so‐called paleo‐endemics, relicts of formerly widespread taxa that declined due to late‐Cenozoic climate changes (Crisp & Cook, 2011; Eiserhardt, Borchsenius, Plum, Borchsenius, Plum, Ordonez, & Svenning, 2015), surviving the Quaternary glaciations in mountain ranges of southern China (López‐Pujol, Zhang, & Ge, 2006). Paleo‐endemics are represented by both angiosperms and gymnosperms in China. Globally, gymnosperms represent one of the most vulnerable groups of living species with almost 40% of all species threatened according to the International Union for Conservation of Nature (IUCN) Red List (Brummitt et al., 2015; Fragnière, Bétrisey, Cardinaux, Stoffel, & Kozlowski, 2015), and China is host to more than 10% of the most threatened evolutionary distinct species (so‐called EDGE species) of this group (Forest et al., 2018). China thus constitutes a global hotspot for gymnosperm diversity, with ~250 species and many threatened, relict lineages such as *Ginkgo biloba*, *Cathaya argyrophylla*, and *Taiwania cryptomerioides* (Eiserhardt, Borchsenius, Sandel, Borchsenius, Sandel, Kissling, & Svenning, 2015; López‐Pujol et al., 2006; Qian, 2001).

Historically, human activities such as land‐use intensification and unsustainable exploitation of resources have been associated with local and regional extirpations of plant species in China (Feng, Mao, Benito, Swenson, & Svenning, 2017; Sun, Zhou, Zhang, & Chen, 2011). One example of this is the extinction of fir (*Abies*) in the Liupan Mountains in the past 2,000 years (Sun et al., 2011). In this case, regional climatic changes most likely caused a decrease in fir‐tree numbers before 2,200 years BP, after which human activities such as fire and logging likely caused a further decrease leading to very small, fragmented populations that were highly sensitive to disturbances, eventually going extinct. Xiang (2001) in Sun et al. (2011) reports that a number of *Abies* species in China have similarly small, fragmented populations, and as their distribution patterns are influenced by the interaction of climate with human activities, these species are especially vulnerable to extinction (Sun et al., 2011). Likewise, Tang et al. (2011) found the remaining population of the relict conifer *Metasequoia glyptostroboides* in China to be detrimentally affected by human activities.

**Figure 1:**
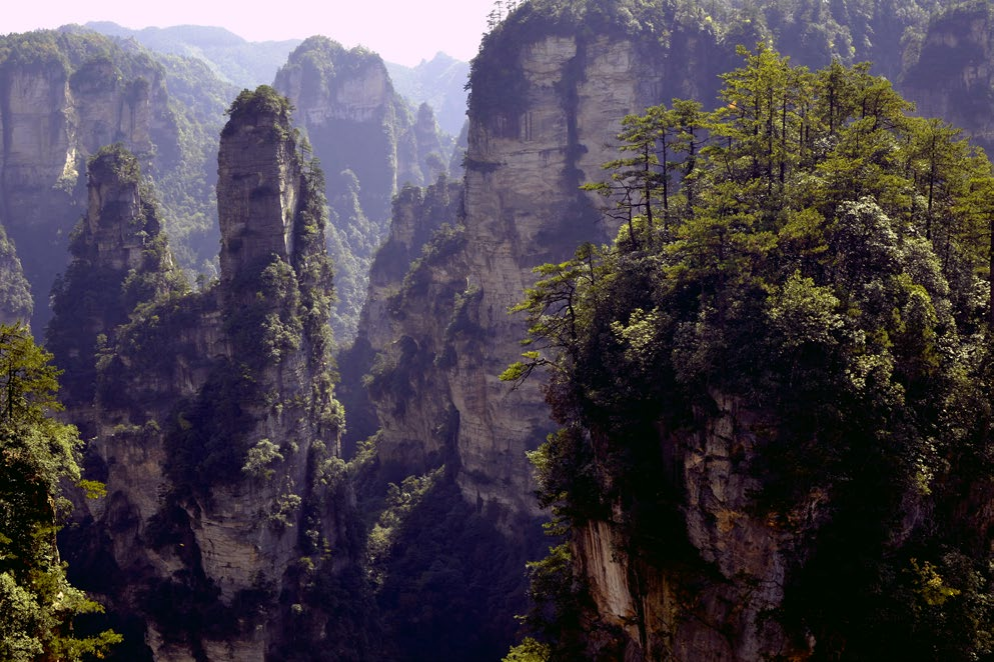
Pine trees (*Pinus* sp.) growing on steep mountains in Zhangjiajie National Forest Park, Hunan Province, China (Photo by D. A. Jensen)

Steep topography may buffer species against anthropogenic pressures (Sandel & Svenning, 2013; Silva, Metzger, Simões, & Simonetti, 2007). Recently, forest cover in China was found to be shaped by topographic slope and this association was affected by human population density, so that in areas with higher human population density, the association of forest cover with steep slopes was higher than in areas of lower human population density (Nüchel, Bøcher, & Svenning, 2019). Such an interactive effect has also been observed for mammals in China (Li, Pan, & Oxnard, 2002; Li et al., 2015), and it is reasonable to suspect that a similar effect exists for Chinese gymnosperm distributions. In fact, relict tree species in China may be constrained to regions of rugged topography not just from its protection against anthropogenic habitat loss and overexploitation, but also because such areas have persistent long‐term suitable conditions on deeper time scales (Feng, Mao, Sandel, Swenson, & Svenning, 2016; Tang, Ohsawa, & Yang, 2014) (Figure 1). An example of such threatened relict species is the paleo‐endemic relict conifer *Cathaya argyrophylla* that currently only exists on the rugged, steep slopes of Mt Bamian and Mt Jinfo in southern China (Tang et al., 2014; Xie, Chen, Jiang, Huang, & Zhu, 1994).

Based on the relationship between human population density and topography (Sandel & Svenning, 2013), we developed a hypothesis for explaining the distribution of gymnosperms in a human‐dominated country such as China. Most species rely on natural habitats for their survival and cannot survive in intensely used agricultural and urban areas. In regions within the natural range of a species, a certain area of natural habitat exists where the species’ ecological requirements are met. The amount of natural habitat in a region is determined by the intensity of human activity in that region, and therefore more natural habitats are available in regions of low human activity compared to regions of high human activity. The natural habitats found in regions of low human activity are distributed more or less equally irrespective of topography. In regions of medium human activity, however, remnant natural habitats predominantly occur in areas of steep topography that are of limited utility for human use. In contrast, in regions of high human activity, land use is intense irrespective of topography and the locations of any remnant patches of natural habitat may be largely determined by societal contingencies. Based on this hypothesis, we expect species that occur in regions of medium human activity to be more associated with steep topography than species in regions of low or high human activity. Further, we expect human‐sensitive (rare) species to show a stronger association with steep topography than common species, given their stronger dependence on natural habitats.

In the present study, we focus on how the interaction between environmental and human factors shapes the distribution of gymnosperm species in China. We are especially interested in the potentially interacting effects of topography (habitat variation, mountain ranges, etc.) and human factors (population density, current and historic) on gymnosperm species distributions. Using species distribution models (SDMs) to analyze the relative importance of human and environmental factors, we address the following hypotheses: (a) Rare, threatened species are more strongly associated with areas of steep terrain than common, nonthreatened species; and (b) if this association with steep terrain is human‐induced, we expect to see a stronger association with steep topography in areas of medium human population densities compared to areas of low and high human population densities.

## 2 METHODS

### 2.1 Species distributional data

The species data were obtained from the *Chinese Vascular Plant Distribution Database*. The database has been assembled over the last 10 years by the Biodiversity and Biosafety Group at the Institute of Botany, Chinese Academy of Sciences. The database consists of native species occurrence records collected in the period 1900–present time, with the majority of collections made after 1950, from both museum inventory reports and published literature such as floras and checklists at a national, provincial, and regional level (Huang et al., 2011). Standardization of taxonomic names in the database was done according to *Catalogue of Life China* (Checklist 2015, http://www.sp2000.org.cn/) and *Flora of China* (http://foc.eflora.cn/). For this study, only gymnosperm species were considered and the dataset used here contains 243 species at species and subspecies level (covering all Chinese gymnosperms). Species occurrences are represented at the county level (2,380 counties), and each occurrence is considered a presence. In order to use these occurrences in species distribution modeling, the centroid of each county polygon was selected as species presence point data. To avoid uncertainties of using the centroid of each county as the presence of the species, counties with an area larger than 10,000 km^2^ were excluded leaving 2,212 counties for further analysis, and consequently excluding much of the large desert areas in the northwestern part of China. In the first analysis, all species were considered, but prior to the species distribution modeling process, species with <10 occurrences were excluded to enhance the quality of model predictions, leaving 163 species for modeling.

### 2.2 Explanatory variables

Climate data were obtained from the bioclimatic variables available at WorldClim (Hijmans, Cameron, Parra, Jones, & Jarvis, 2005), where a set of temperature and precipitation data was chosen based on the importance for year‐round tree survival. Mean annual temperature, with the bioclimatic variable code BIO1, and annual precipitation (BIO12) were chosen based on the common use of these data in describing species distributions on a large scale. Minimum temperature (BIO6), temperature seasonality (BIO7), and precipitation of driest month (BIO14) were chosen based on the belief that they are more directly linked to species survival; for example, the minimum temperature is more likely to represent an environmental limitation for tree growth than the annual mean. Temperature seasonality was calculated as the annual range in temperature between the maximum value of the warmest month and the minimum value of the coldest month.

Furthermore, a dataset containing elevation was gathered from WorldClim as well. Topographic range (TR) was estimated as the maximum difference in elevation within each county and used as a proxy for steep terrain (a larger TR equals steeper terrain) in the further analyses. To ensure congruence among the topographic range variable and other steepness indicators, analyses were performed comparing topographic range values with both topographic maximum values as well as standard deviation (*SD*) of the topographic range in each county. The Spearman correlations were >0.90 (range ~ maximum = 0.93, range ~ *SD* = 0.98), supporting the use of topographic range as a proxy for steep terrain. All the environmental data had a resolution of 30 arc seconds (~1‐km^2^ grid cells at the equator).

Human influence variables included population density estimates, one current (2015CE) and three historic (2000BCE, 0CE, and 1100CE) (Klein Goldewijk, Beusen, Van Drecht, & De Vos, 2011), as well as a human influence index (Hii). Despite human population data from 1975 CE being a better temporal match for the species occurrence data, we chose to use the 2015 CE human population density variable, since it is highly correlated with 1975 CE (Spearman's *r* = 0.97), and it likely has a higher accuracy as well.

The human influence index is constructed from several human‐related data layers: human population density, built‐up areas, nighttime lights, land use/land cover, coastlines, roads, railroads, and rivers; and provides a score from 0 to 72 where 0 represents no human influence and 72 the maximum of human influence (Sanderson et al., 2002). The human population density data were retrieved from the HYDE database (HYDE v3.1, http://themasites.pbl.nl/tridion/en/themasites/hyde/basicdrivingfactors/population/index-2.html), while the human influence index was obtained from the “Last of the Wild v2” collection (http://sedac.ciesin.columbia.edu/data/set/wildareas-v2-human-influence-index-geographic).

Variables were processed prior to modeling to standardize data to the same projection, grid cell size, and spatial extent and to compute the topographic range variable. Standard procedures (Zonal statistics) were used to combine the datasets into a resolution matching the coarsest dataset (county scale) by computing the mean of each variable within each county. To avoid multicollinearity problems, the final set of variables for the analyses was selected based on variance inflation factor (VIF) values, using a threshold of 5. The selected variables were temperature seasonality (BIO7), precipitation of driest month (BIO14), topographic range, population density at 0CE, population density at 2015CE, and human influence index (Hii) (Table 1).

**Table 1:** Variable names used for analyses

Variable explanation	Variable name
Temperature seasonality (BIO7)	TempSeason
Precipitation of driest month (BIO14)	PrecipDM
Topographic range	TR
Human population density at 0CE	HPDHist
Human population density at 2015CE	HPDCur
Human influence index	Hii

### 2.3 Introductory analysis

#### 2.3.1 Species association with topography and human population density

To test the first hypothesis, that is, that rare species are on average more associated with steep terrain than common species, we split the species into three groups: very rare species (VR), rare species (R), and common species (C) based on species occurrence (Table 2). Within each group, we estimated the mean topographic range and the mean human population density for each species as the mean of that variable of all the counties where the species is present. A nonparametric analysis of variance (Kruskal–Wallis test) as well as a Wilcoxon test (Wilcoxon signed rank) was performed in order to compare group means and determine groups that are significantly different.

**Table 2:** Species groups considered in the introductory analyses

Group	Explanation	*N* species	*N* counties
Very rare (VR)	Species occurrence ≤ 10	80	179
Rare (R)	Species occurrence 11–99	84	703
Common (C)	Species occurrence 100–787	77	2006

Notes

Group = name of each group; Explanation = an explanation for the division of the groups; *N* species = the number of species in the group; and *N* counties = the number of counties that are covered by the group.

### 2.4 Interacting effects of topography with human factors

#### 2.4.1 Species distribution modeling

All species distribution modeling was conducted using the maximum entropy algorithm (MaxEnt) within the “biomod2” package in R (Thuiller, Georges, Engler, Georges, & Thuiller, 2016). We chose MaxEnt as it is known to perform well with presence‐only data, does especially well on datasets with few occurrences, and is not affected by locational error in the occurrences (Elith et al., 2006; Graham et al., 2008; Wisz et al., 2008). As our dataset only contains presences of our species, we chose to create pseudo‐absences for each species during the modeling process. Default modeling options as well as a number of fixed settings were used to create all species distribution models. Within the BIOMOD functions, a number of settings can be manually set before modeling. For each model, a defined number (1,000) of pseudo‐absence points was selected using an intrinsic, random strategy within the “BIOMOD_FormattingData” function (Thuiller et al., 2016). This random method of selecting pseudo‐absence data has been shown to provide better models when modeling species distributions (Barbet‐Massin, Jiguet, Albert, & Thuiller, 2012). Further, for each model the data were split with 80 percent of the data used for training the model and 20 percent for testing the model performance. Performance testing was an intrinsic part of the modeling process and we chose to do two performance tests (evaluation runs) per model. Each evaluation run was composed of two runs based on the pseudo‐absence datasets and one run using the full dataset, resulting in three models for each evaluation run and thus six models for each species. From each model, variable importance and model evaluations, area under the curve (AUC), and true skill statistic (TSS) values were extracted. The metric of variable importance is obtained by a simple correlation analysis between reference model predictions and predictions done by using a shuffled version of a given variable. The value of variable importance is then 1 minus the correlation between the reference model predictions and the shuffled variable predictions, where a value of 1 represents the highest importance and a value of 0 represents the lowest importance. Variable importance estimates were averaged for all models having an AUC >0.80 (Thuiller et al., 2005). Response curves were generated for selected variables based on model predictions using the Loess regression method and these plots were then used to support and validate patterns obtained through the variable importance metric.

**Figure 2:**
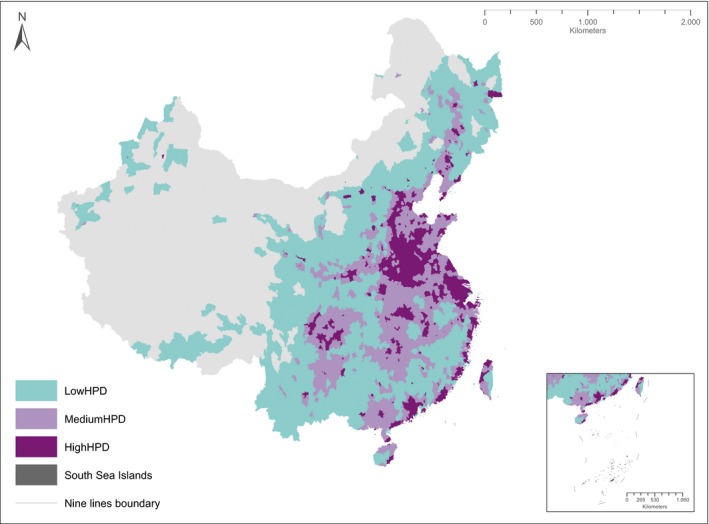
Map of China showing human population zones based on current population density. Only counties with an area less than 10,000 km^2^ were considered. LowHPD = low human population density, which contains human population densities from 0 to 200 persons/km^2^; MediumHPD = medium human population density, which contains human population densities from 200 to 600 persons/km^2^; and HighHPD = high human population density, which contains human population densities from 600 to 1,500 persons/km^2^. Insert shows the islands in the China South Sea. The map projection used here is the Albers Equal Area Conic projection

To address the possible interaction of topographic range with human factors, the explanatory data were split according to zones of human population density (0–200, 200–600, 600–1500 persons/km^2^; Figure 2), that is, low, medium, and high human population density (LowHPD, MediumHPD, HighHPD), respectively. We chose the cutoffs between the zones in order to represent the extremes in human population density better, as the aim of our study is to investigate the effect of human activity. This means that we made conservative cutoffs for the low‐ and high‐human‐population‐density zones, avoiding urban areas in the LowHPD zone while including only densely populated areas in the HighHPD zone. One species distribution model was created for each species for each zone. Not all species were present in all zones, so species that were only present in a single zone were excluded. Species‐specific differences were explored by grouping variable importance estimates according to species IUCN status, reflecting their sensitivity to human influence and climate change. Two groups were identified: nonthreatened (all species assessed as least concern, LC; or near threatened, NT) and threatened (all species assessed as vulnerable, VU; endangered, EN; or critically endangered, CR). For each group of species, the variable importance estimates were compared between the three human population density zones, and ANOVAs (Tukey's HSD method) were carried out to separate the zones according to significance levels. Response curves were generated for each of the two species groups as well as for each individual species within the threatened species group. To explore if current Chinese land‐use policies regarding topography have evident effects in our data, we generated a new slope variable within each county (maximum slope) and plotted this variable against the model predictions.

To strengthen the analysis and for validating patterns within the results, a set of species were selected based on their relation to human activity, forming two groups that we named human‐sensitive and human‐promoted species. The human‐sensitive species group contains *Abies ernestii* and *Taxus wallichiana*, which are both slow‐growing, late‐maturing tree species with populations that are affected by human influence such as logging and other nonsustainable use (Thomas & Farjon, 2011; Xiang & Rushforth, 2013). The human‐promoted species group contains *Pinus massoniana* and *Cunninghamia lanceolata,* two species that historically have been favored by humans and thus are widely planted for forestry purposes in plantations, in parks, and on temple grounds (Dou et al., 2013; Farjon, 2013; Kuang, Sun, Wen, Zhou, & Zhao, 2008; Li & Ritchie, 1999; Xiang, Christian, & Zhang, 2013). Response curves for these species were used for validating the patterns of variable importance of the two IUCN groups, threatened and nonthreatened.

## 3 RESULTS

### 3.1 Introductory analysis

**Figure 3:**
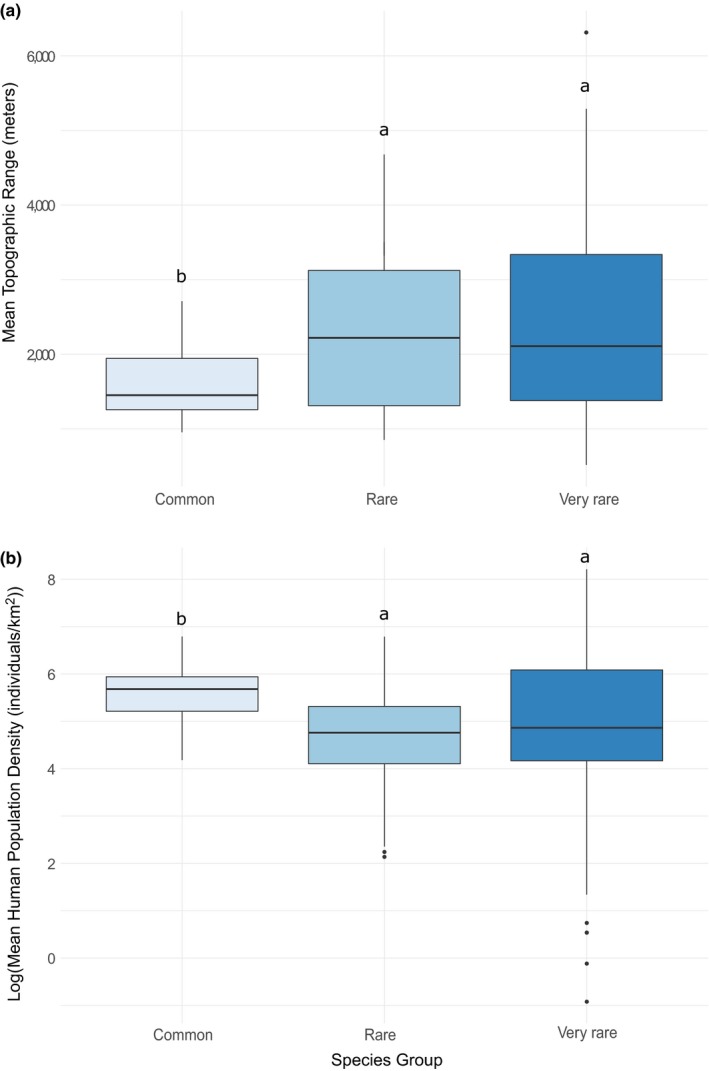
Boxplots representing mean values of species in three groups: very rare, rare and common species. Panel (a) shows the distribution of species means of topographic range (TR) for the three groups. Panel (b) shows the distribution of species means of human population density (HPD) on a logarithmic scale for the three groups. Letters above boxes represent significant differences in mean values among the groups; that is, identical letters represent similar means and dissimilar letters represent different means, according to a Wilcoxon rank‐sum test (Table 3)

Very rare and rare species were found to generally occur in areas with steeper slopes and lower human population densities than common species (Figure 3, Table 3).

**Table 3:** Results of the pairwise comparison tests (Wilcoxon rank‐sum test)

Pairwise comparisons, Wilcoxon rank‐sum test		
	Common	Rare
*Topographic range*		
Rare	**0.0029**	–
Very rare	**0.0066**	0.8827
*Current human population density*		
Rare	**2** × 1**0^–7^**	–
Very rare	**0.0046**	0.4609

Notes

Top: Results from the first test comparing mean topographic range between species groups. Bottom: results of the second test comparing mean human population density between species groups. Group names are explained in Table 2. Numbers in bold show significant (*p* < .05) difference between groups.

### 3.2 Interacting effects of topography with human factors

After eliminating all models with AUC values below 0.8, the mean of the AUC values of the models used for analyzing the hypotheses were all above 0.9, while the mean of the associated TSS values were all above 0.79 (Table 4).

**Table 4:** AUC and TSS values of the models selected for further analyses

Dataset	Number of models	AUC (*SD*)	TSS (*SD*)
LowHPD	134	0.924 (0.038)	0.801 (0.104)
MediumHPD	89	0.911 (0.050)	0.792 (0.142)
HighHPD	47	0.905 (0.044)	0.799 (0.112)
All data	145	0.926 (0.043)	0.798 (0.121)

Notes

AUC and TSS values are presented as the mean of the selected models for all species within each dataset. *SD* represents the standard deviation of the mean. LowHPD = low‐human‐population‐density zone; MediumHPD = medium‐human‐population‐density zone; HighHPD = high‐human‐population‐density zone; All data = no zones.

#### 3.2.1 All species

**Figure 4:**
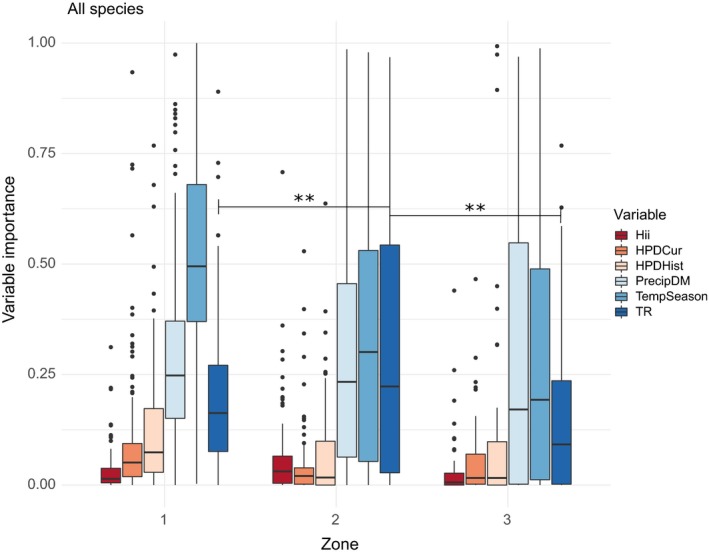
Variable importance values for all species analyzed by human population density zone. Zone 1 = low‐human‐population‐density zone; zone 2 = medium‐human‐population‐density zone; and zone 3 = high‐human‐population‐density zone. Number of species in zone 1 = 129; number of species in zone 2 = 90; and number of species in zone 3 = 47. For variable names explained, see Table 1. Significance of the topographic range variable between the zones is displayed using the following significance levels: ns = *p* > .05, * = *p* ≤ .05, ** = *p* ≤ .01, *** = *p* ≤ .001

Topographic range had a higher importance as a predictor of species occurrence at medium human population density than at low or high human population density (Figure 4, Table 5). Contrastingly, the human influence variables, current human population density and historic human population density had significantly lower importance at medium human population density compared to low human population density, with the human influence index variable showing an opposite relationship (Figure 4, Table 5).

**Table 5:** Results from ANOVA tests performed to explore differences in variable importance estimates obtained from SDM modeling

Zone	Group	*p*‐value					
		TR	TempSeason	PrecipDM	HPDCur	HPDHist	Hii
L vs. M	All species	**1.86** × **10^–3^**	**1.5** × **10^–4^**	0.918	**0.006**	**0.020**	**0.008**
	Nonthreatened	0.469	**0.020**	0.502	**0.011**	0.083	**0.007**
	Threatened	**9.14** × **10^–4^**	**8.79** × **10^–3^**	0.78	0.441	0.203	0.452
L vs. H	All species	0.562	**3.87** × **10^–4^**	1.00	0.196	0.999	0.892
	Nonthreatened	0.528	**0.007**	0.972	0.343	0.994	0.839
	Threatened	0.956	0.155	0.923	0.531	0.587	0.53
M vs. H	All species	**1.08** × **10^–3^**	0.857	0.958	0.749	0.107	0.153
	Nonthreatened	0.128	0.726	0.778	0.574	0.172	**0.010**
	Threatened	**0.016**	0.959	0.996	0.978	0.973	0.975

Notes

SDMs were fitted for each species group (all, nonthreatened, and threatened) within each zone (L, M, H). P‐values from the ANOVA tests are reported here. Bold indicates *p* < .05. Zone = zones compared; for example, zone L versus M compares the low‐human‐population‐density zone with the medium‐human‐population‐density zone. Zone L = low human population density; zone *M* = medium human population density; and zone H = high human population density. For variable names explained, see Table 1.

#### 3.2.2 Nonthreatened versus threatened species

**Figure 5:**
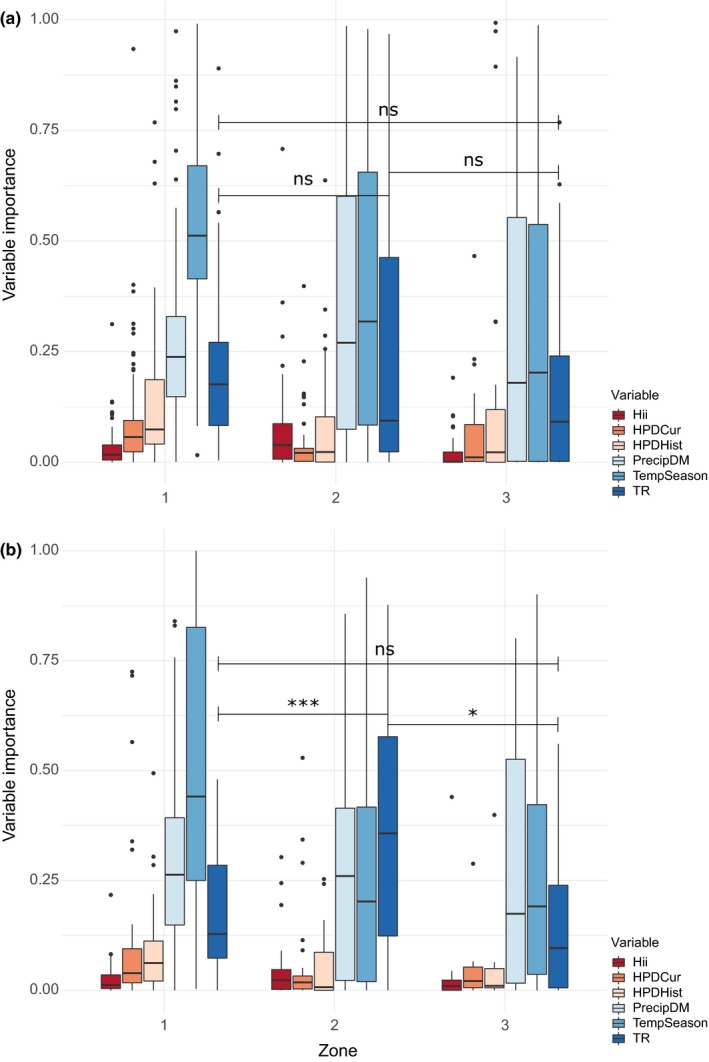
Panel (a): Variable importance values for the nonthreatened species analyzed within zones of human population density. Panel (b): Variable importance values for the threatened species analyzed within zones of human population density. Zone 1 = low‐human‐population‐density zone; zone 2 = medium‐human‐population‐density zone; and zone 3 = high‐human‐population‐density zone. For variable names explained, see Table 1. Significance of the topographic range variable between the zones is displayed using the following significance levels: ns = *p *> .05, * = *p* ≤ .05, ** = *p* ≤ .01, *** = *p* ≤ .001

**Figure 6:**
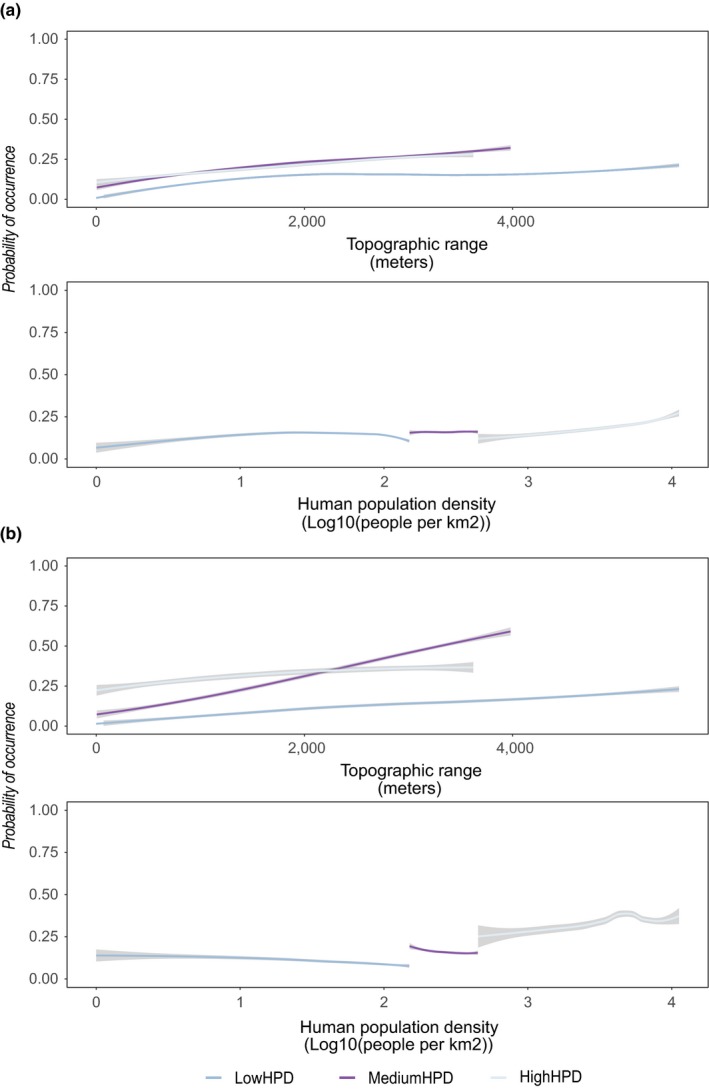
Response plots from maximum entropy (MaxEnt) models for the topographic range and the current human population density variables in the three human population density zones. LowHPD = low‐human‐population‐density zone; MediumHPD = medium‐human‐population‐density zone; and HighHPD = high‐human‐population‐density zone. Panel (a) shows response plots for the group of nonthreatened species while panel (b) shows response plots for the group of threatened species. Regression lines display the mean (± 1STD) response of all the species in the zone using the Loess regression method

Considering the nonthreatened species alone, the importance of topographic range was not significantly different between the human population density zones (Figure 5a, Table 5). Response plots for the topographic range variable however, showed a general positive response in all three zones, with the probability of species occurrence increasing with increasing topographic range (Figure 6a). The two human influence variables current human population density and human influence index showed a significant difference in variable importance between the low‐ and medium‐human‐population‐density zones (Figures 5a and 6a, Table 5).

**Figure A1:**
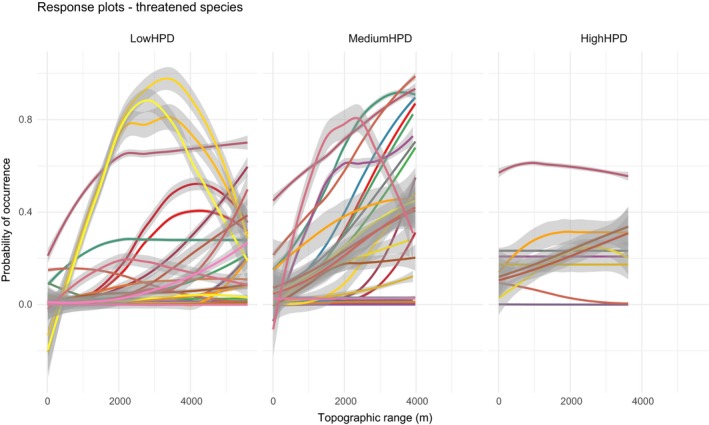
Response plots from maximum entropy (MaxEnt) models for each species in the threatened species group within each human population density zone. Colors represent individual species. LowHPD = low‐human‐population‐density zone; MediumHPD = medium‐human‐population‐density zone; and HighHPD = high‐human‐population‐density zone. Regression lines display the mean (± 1STD) response using the Loess regression method

**Figure A2:**
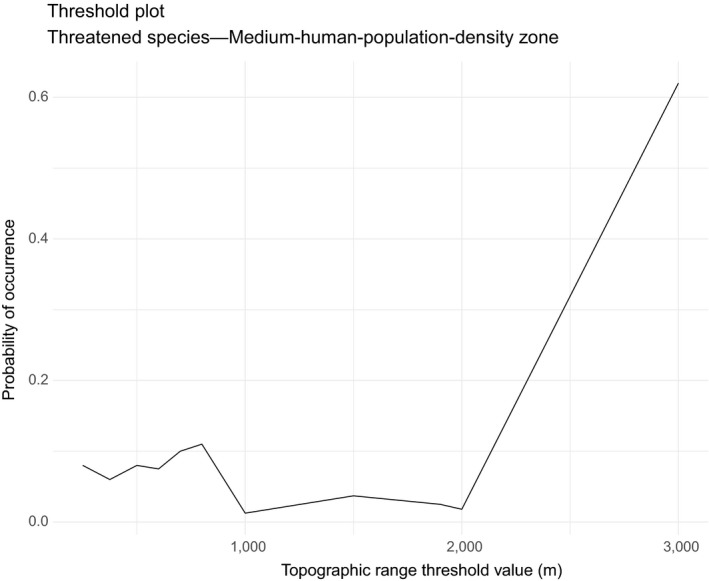
Threshold values from individual species response curves as estimated visually. 11 out of the 37 species in the threatened species group exhibited this response. Values on the x‐axis represent threshold values of topographic range for each of 11 threatened species. Values on the y‐axis are the associated probability of occurrence obtained from the original response curves

In contrast, considering only the threatened species, the importance of topographic range was significantly higher at medium human population density compared to low and high human population density (Figure 5b, Table 5). Similarly, the response plot for the topographic range variable showed a marked positive increase in probability of occurrence with increasing topographic range (steepness) in the medium‐human‐population‐density zone (Figure 6b). All human influence variables showed low variable importance, and we found no significant differences between the zones for any of them (Table 5, Figures 5b and 6b). Response plots for the topographic range variable, for each individual species in the threatened species group showed that at least some species have a threshold‐like response to topography and the linear trend that was seen for this group overall (Figure 6b) at least partially is a product of averaging across these individual species thresholds (Figures A1 and A2).

**Figure 7:**
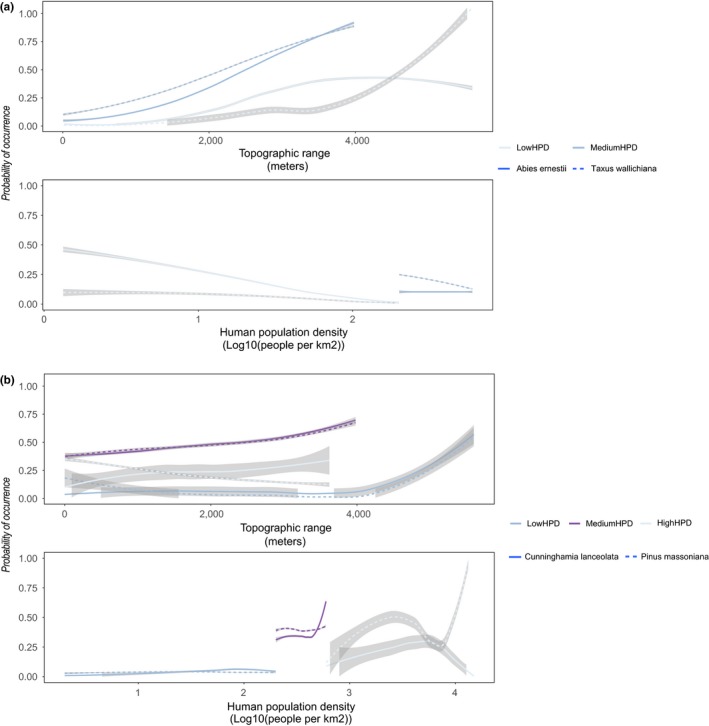
Response plots from maximum entropy (MaxEnt) models. Panel (a): human‐sensitive species, *Abies ernestii* and *Taxus wallichiana*. The species only occurred in two of the three human population density zones. Colors denote zones: light blue = low‐human‐population‐density zone; and dark blue = medium‐human‐population‐density zone. Line type denotes species: hard line = *Abies ernestii*; and broken line = *Taxus wallichiana*. Panel (b): human‐promoted species, *Pinus massoniana* and *Cunninghamia lanceolata*. Colors denote zones: dark blue = low‐human‐population‐density zone; dark purple = medium‐human‐population‐density zone; and light blue = high‐human‐population‐density zone. Line type denotes species: hard line = *Cunninghamia lanceolata*; and broken line = *Pinus massoniana*. Regression lines display the mean (± 1STD) response using the Loess regression method

**Figure 8:**
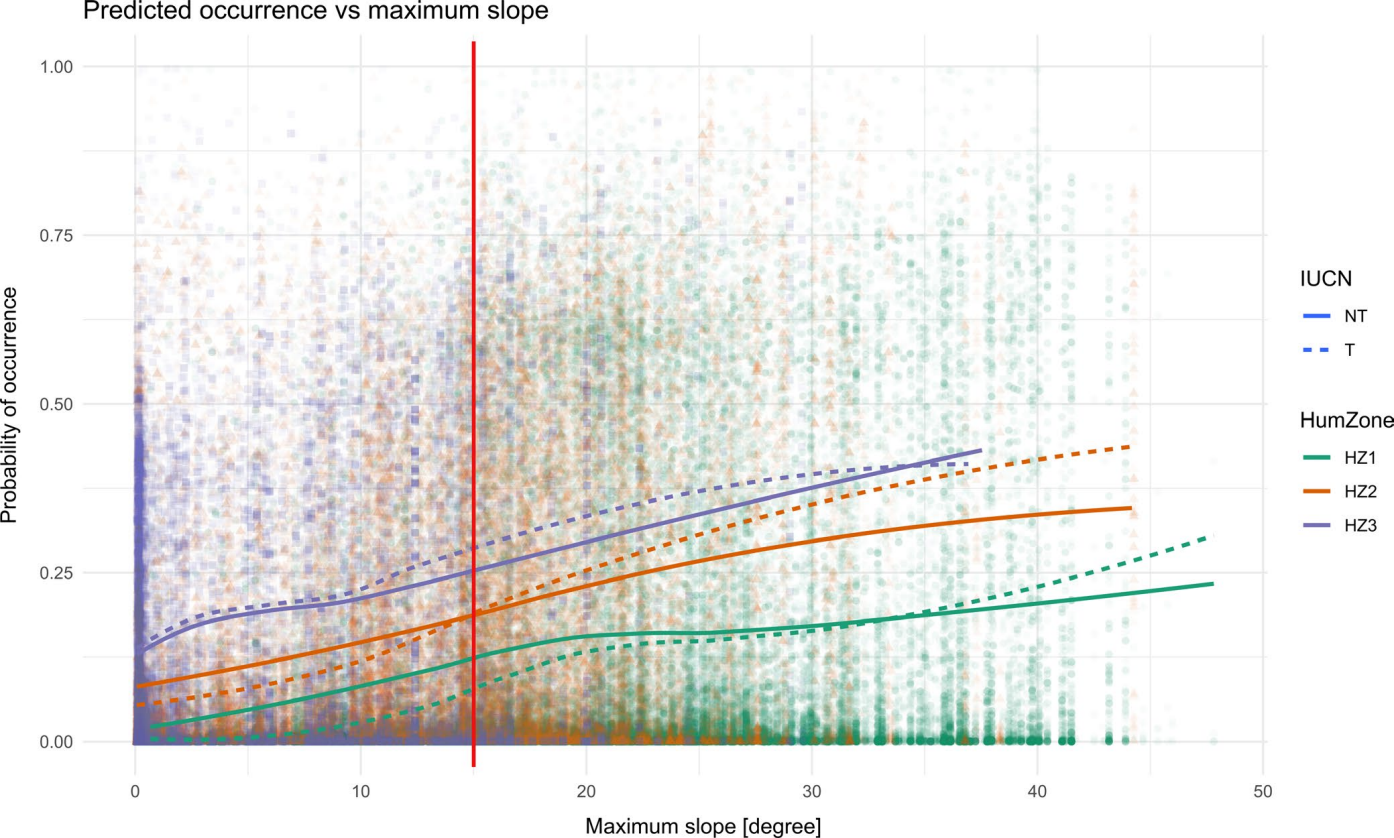
Plot showing the MaxEnt model predictions plotted against the maximum slope value of each county. Regression lines show the mean (± 1STD) and were generated using the Loess method for both threatened (T) and nonthreatened (NT) species in each of the human population density zones. Line type denotes species group. Color denotes human population density zone where HZ1 = low‐human‐population‐density zone; HZ2 = medium‐human‐population‐density zone; and HZ3 = high‐human‐population‐density zone. The background points represent all species within all three groups (colors are the same as for the lines). The red vertical line indicates slope = 15 degrees

The plot generated to assess if there are evident effects of Chinese land‐use policies (stating that slopes >15° are unsuitable for cultivation) on gymnosperm species occurrences shows that for all zones and for both species groups, there is an increase in the probability of occurrence with increasing slope values. For species in the medium‐human‐population‐density zone, there is a shift in the probability of occurrence for the two groups at a slope value of approximately 15°, so that the threatened species are more probable to occur above this slope value (Figure 8), that is, as expected from the initial hypothesis that rare, threatened species are more strongly associated with steep terrain than common species.

#### 3.2.3 Validation of results using human‐sensitive and human‐promoted species

We found a strong positive response to topographic range, with the strongest relationships for the human‐sensitive species as well as for the human‐promoted species *C. lanceolata* in the high‐human‐population‐density zone (Figure 7a,b).

Response plots for the human‐sensitive species show a positive response to topographic range especially in the medium‐human‐population‐density zone, while the response to human population density is generally negative or neutral (Figure 7a). Response plots for the human‐promoted species show a mixed response to topographic range, generally neutral, but turning increasingly positive at high values of topographic range (Figure 7b). The responses to human population density range from neutral in the low‐human‐population‐density zone, a strong positive response for *Cunninghamia lanceolata* in the medium human population density, to a strong positive response for *Pinus massoniana* in the high‐human‐population‐density zone (Figure 7b).

## 4 DISCUSSION

In this study, we used a common SDM method to investigate how environment and human activity interact in shaping gymnosperm species distributions in China. We were specifically interested in the interaction between steep topography and human activity, as these have previously been found to interact in, for example, shaping forest cover and the distribution of certain mammal species. The specific hypothesis that we wanted to test was that species (especially threatened or rare) are more associated with a steep topography in regions of medium human activity because a large amount of natural habitat exist here but only in places of limited human activity, that is, on steep slopes. From the results, we find that rare species occur more often in areas with high topographic range and low human population density compared to common species. Our results further show that human activity does shape species distributions through interactions with topography, with a stronger importance of topography at medium human population densities, especially for threatened species, showing that steep terrain provides partial refuge against human pressures.

### 4.1 Species association with topography and human population density

Results from the introductory analyses addressing the question about species association with topography and human population density revealed that very rare and rare species are generally found in areas of higher topographic range and lower human population density than common species (Figure 3). This finding, that rare species are more often found in areas of higher topography, is supported by the literature where rare species often occur in habitats that are unsuitable for human use, such as steep slopes in mountain areas (Lavergne, Thuiller, Molina, & Debussche, 2005; Zhang & Ma, 2008). However, we cannot simply conclude that rare species are just naturally occurring in mountain areas nor that they have been constrained to these areas by human activity. Some rare species are considered naturally associated with certain habitats, although at least some of these species display this association because of historic anthropogenic activities as opposed to more natural causes (Cromsigt, Kerley, & Kowalczyk, 2012). However, it is not within the scope of this study to distinguish the cause of the association of rare species with topographic range, but rather to investigate the interaction of this association with human activity.

### 4.2 Interacting effects of topography with human factors

Results from the analysis addressing the possible interaction of topography with human activity, show that species in general and threatened species specifically, are more associated with steep topography in areas of medium human population densities (Figures 4 and 5b). Although there is no significant difference in importance of topographic range across the zones for the nonthreatened species group, response plots for both groups show a positive relationship with topographic range (Figure 6), suggesting that steep topography provide some level of buffering against human pressures for threatened and nonthreatened gymnosperm species alike. These findings support our hypothesis that human activity creates a landscape of patchy natural habitats that leave most room for especially threatened species in areas of steep topography in regions of medium human activity. In the regions of low human activity, natural habitats exist across the entire topographic range, and species are able to occupy their optimal habitat. In regions of high human activity, human land use is intense regardless of topography, and natural habitats exist in patches within this matrix of human land use so that species are forced to occupy any available habitats. In these cases, we did not expect to see a high association with mountain ranges, and our results support this as the importance of topographic range is significantly lower in the low‐ and high‐human‐population‐density zones compared to the medium‐human‐population‐density zone (Figures 4 and 5b). In the regions of medium human activity, however, natural habitats exist in largest amounts where conditions do not allow for human utilization, for example, steep slopes. In this case, we expected to see a high association of species occurrence with topography, and our results do show a higher importance of topography in the medium‐human‐population‐density zone for all species in general and threatened species specifically (Figures 4 and 5b).

It has previously been established that human activities have had a direct impact on species distributions in China. For example, range contractions and extinctions of certain Chinese mammals have been linked to the expansion of the human population as well as intensified human activities (Li et al., 2015; Zhao, Ren, Garber, Li, & Li, 2018). These findings support the idea that anthropogenic pressures have the ability to shape species distributions. In this study, we find that species, especially threatened species, display a higher association with topographic range at medium human activity. An association with steep topography is not necessarily a human‐induced effect though, since rare species are often naturally associated with mountain ranges (Sandel et al., 2011). However, given the interaction of the topographic effect with threat status and with human population density, it is likely that some of these species that are thought to naturally occur in mountain ranges/steep slopes could really be so‐called refugee species (Kerley, Kowalczyk, & Cromsigt, 2012). Refugee species are species that have been forced into marginal habitat by historical human activity. From their current range, these species might seem to prefer a certain kind of habitat, although their performance here may be suboptimal. In reality, these species might perform better in other habitats, but to identify their natural preferences requires historical data and experimental approaches (Cromsigt et al., 2012). Chinese examples of such species include the giant panda (*Ailuropoda melanoleuca*) and the snub‐nosed monkeys (*Rhinopithecus*), all of which are considered to be constricted to their current ranges because of historical human activities (Han et al., 2019; Nüchel, Bøcher, Xiao, Zhu, & Svenning, 2018; Wei et al., 2015).

In terms of our study, it seems that there are two scenarios that can lead to species having a strong association with topographic range – one natural and one human‐induced. If it were natural, we would not expect to see the strength of this association change across the human population density zones. On the other hand, if the association were human‐induced, we would indeed expect to see an interaction of human activity with this association as we do in this study (Figures 4 and 5b). A recent study finds a similar interaction between slope and tree cover that is affected by human population density (Nüchel et al., 2019). Here, the authors suggest that steep slopes might act as refuge for forests since land use in these areas is less intense. Another study on Chinese gibbons finds an association with topography, similar to our study, where populations of gibbon persisted longer at higher elevations and disappeared earlier from the northern and eastern parts of China, consistent with the demographic expansion of humans (Turvey, Crees, & Di Fonzo, 2015). Results from our study similarly suggest that especially threatened species are strongly associated with steep topography, and because of the interaction with human population density, this association is possibly human‐induced or exacerbated. Human land use is central to the degradation of natural habitats (Foley et al., 2005) and it is reasonable to expect to see effects of human land use on Chinese gymnosperm species association with topography. Studies on Chinese land use in hilly regions, suggest that farmland in sloped terrain >15° should be abandoned due to increased soil erosion and nitrogen loss (Dong, Liu, & Shi, 2010; Fu et al., 2004). Evaluating model predictions from our study against maximum slope in each county, suggest that threatened species, more often than nonthreatened species, find refuge on steep slopes where human cultivation is advised against (Figure 8). This result further supports the theory that the association of threatened gymnosperm species with steep topography is indeed related to human activity.

To validate the patterns described above, we chose specific species known to be either sensitive to human activity or promoted by human activity to represent the threatened versus the nonthreatened species groups. Response plots for the human‐sensitive species show a positive response to topographic range especially in the medium‐human‐population‐density zone supporting our idea of species being more associated with steep areas here. Further, both species show neutral or negative responses to human population density in both zones likewise supporting this idea (Figure 7b). In contrast, we found a strong positive response to human population density in the medium‐ and high‐population‐density zones for *C. lanceolata* and *P. massoniana*, respectively (Figure 7b). A response like this is in line with these species being favored by human activities, having often been used in reforestation projects aimed at, for example, carbon sequestration and soil‐erosion mitigation, or planted in parks and temples. Indeed, large parts of the afforestation and reforestation efforts in China have been focused on the hilly southern regions, where soil erosion following forest logging has been a major problem (Zheng et al., 2008).

The resolution of data used for modeling species distributions is important to consider. Depending on the specific question that is addressed, the resolution needs to match as changes in grain size can influence the patterns of species presences (Guisan, Graham, Elith, & Huettmann, 2007). The use of coarse‐grained data when modeling species distributions has in many cases proved to yield similar model performances as using finer grained data. In a study investigating the influence of grain size on the performance of species distribution modeling, the authors found that a 10‐fold coarsening of the grain size did not have a substantial effect on model performance (Guisan et al., 2007). Likewise, another study looked at the effect of increasing grain size on the size and location of predicted species distributions, and found that changes in resolution below 16‐fold only slightly affected model performance (Seo, Thorne, Hannah, & Thuiller, 2009).

The use of coarse‐grained species occurrence data could potentially limit the ability of the SDMs to capture the range of each explanatory variable and convey more fine‐scale topography‐ and climate‐related microhabitats that are important for especially threatened plant survival and persistence (Austin & Van Niel, 2011). However, several studies have found that the effects of topography operate at regional to continental scales and at coarse grain sizes. One study found that topographic indices better explained *Rhododendron* richness at coarse scale (grain size >1°), while another study similarly found that bird species richness was better explained at coarse grain sizes (40 and 80 km resolution) owing to climate, topography and human land use operating on these scales (Yamaura, Amano, Kusumoto, Nagata, & Okabe, 2011; Yu et al., 2015). Likewise, a recent study found that topography acted as a refuge for wolves (*Canis lupus*) at several spatial scales (Grilo et al., 2019). As the choice of grain size depends on both the available species and environmental data and the modeling objectives, we argue that using a county‐level resolution in this study matches the study question since we expect to see the effects of topography at this landscape level.

## 5 CONCLUSIONS

The results of this study suggest that gymnosperm species in China in general, and threatened species specifically, are affected by human activity in such a way that they are more often found in habitats of steep topography in regions where human activity is at medium level, in line with a buffering effect of steep topography against human pressures up to moderate intensity of land use, allowing for natural habitat to exist primarily in mountain areas. Many of the Chinese gymnosperm species are vulnerable to human impacts and risk extinction, and conservation measures should be undertaken to secure the future existence of these species in their natural habitats. However, caution should be taken to determine if the current habitat of the species is in fact optimal for the species in question or if the species qualifies as a refugee species and its current range is instead a result of historical human activity. To determine this with certainty requires further studies into individual species performance in their current habitats (Cromsigt et al., 2012; de Medeiros, Hernández‐Lambraño, Ribeiro, & Sánchez Agudo, 2018). One specific conservation measure to consider is the introduction of threatened gymnosperm species into reforestation programs, thus establishing new populations in areas where deforestation has been the major issue. For a long time, reforestation programs have focused on planting species‐poor stands of trees with fast growth and high yield, such as non‐native species of *Populus* and *Eucalyptus*, but the effect is only short‐lasting (Cao et al., 2011; Hua et al., 2016; Liu, 2008; Stone, 2009). Using native species in a mixed‐forest setting instead could possibly contribute to a longer lasting effect as well as provide higher economic and ecological benefits and at the same time, it would serve to conserve species distributions in their native range (Hua et al., 2016; Stone, 2009). Because of limited enforcement of conservation measures in China's currently protected areas, species are under a constant threat of logging and collecting from local rural communities even there (Sang, Ma, & Axmacher, 2011). Therefore, it will also be important to enhance enforcement of protection of already protected areas to avoid losing the remaining populations of threatened species (Pan et al., 2016). Considering ongoing climate change, focus on conservation in areas with varied topography will be vital since such areas better allow for species to track climate (Sandel et al., 2011). For this reason, protection of current‐, along with establishment of new protected areas containing a high degree of topographic heterogeneity should be a central component in the development of systematic spatial conservation planning in China as well as abroad (Ackerly et al., 2010; Suggitt et al., 2018; Theobald, Harrison‐Atlas, Monahan, & Albano, 2015).

## CONFLICT OF INTEREST

The authors have no competing interests affecting the work of this paper.

## AUTHOR CONTRIBUTIONS

All authors designed the study and contributed to the writing. K. Ma provided the data. D.A. Jensen analyzed the data and led the writing.

## Data Availability

Species occurrence data used for building the biomod2 SDMs can be accessed on the Dryad online repository (DOI accession number: https://doi.org/10.5061/dryad.v15dv41s4).

## APPENDIX 1

This appendix contains information regarding the individual response curves generated from the maximum entropy models for each species of the threatened species group. The aim was to investigate the mean trend of this group as is seen in Figure 6b, to identify possible threshold values that were not evident from this mean trend.

As is seen in Figure A1, threatened species display a range of different responses to topographic range. Some species seem to exhibit a threshold in their response that disappears when averaging the responses as in Figure 6b. We did a systematic plot of the threshold values (visually estimated from each response plot) of those species exhibiting this behavior in the medium‐human‐population‐density zone and found that 11 out of 37 species had this threshold‐like response. The systematic plot of these threshold values is shown in Figure A2.
